# Clinical Predictors of Prolonged Hospitalization in Children with Community-Acquired Pneumonia

**DOI:** 10.3390/children13020226

**Published:** 2026-02-05

**Authors:** Nasser S. Alharbi, Fahad Alsohime, Waleed Abdulla Alharthi, Bader A. Alqarni, Afrah Ghawi, Abdulkarim Alrabiaah

**Affiliations:** 1 Department of Pediatrics, College of Medicine, King Saud University, Riyadh 11451, Saudi Arabia; falsohime@ksu.edu.sa (F.A.); alrabiaah@ksu.edu.sa (A.A.); 2 Department of Pediatrics, King Saud University Medical City, King Saud University, Riyadh 11451, Saudi Arabia; 3 Pediatric Hospital, King Saud Medical City, Riyadh 12746, Saudi Arabia; w.alharthi@ksmc.med.sa; 4 Department of Pediatrics, Ad-Diriyah Hospital, Ministry of Health, Riyadh 13717, Saudi Arabia; baahalqarni@moh.gov.sa; 5 Department of Pediatrics, King Fahad Centeral Hospital, Jazan 45142, Saudi Arabia; aaghawi@moh.gov.sa

**Keywords:** pneumonia, length of stay, empyema

## Abstract

**Highlights:**

**What are the main findings?**

• Disease severity, pneumonia complications, and chronic medical conditions independently predict prolonged hospitalization (≥10 days) in 27.5% of children with community-acquired pneumonia.
• Early identification of these predictors enables risk stratification at admission and the implementation of targeted interventions to reduce length of stay.

**What are the implications of the main findings?**

• Recognizing these predictors at admission allows for early risk stratification and more accurate caregiver counseling.
• Quality-improvement projects should focus on streamlining care and implementing tailored pathways to reduce unnecessary hospital delays.

**Abstract:**

**Objectives:** This study aimed to identify key predictors of prolonged hospitalization in children with community-acquired pneumonia by comparing demographic and clinical characteristics between patients with expected and extended hospital stays. **Methods:** A retrospective cohort study was conducted for children younger than 15 years hospitalized with pneumonia between May 2015 and March 2020. Patients with hospital-acquired pneumonia or additional diagnoses were excluded. Demographic and clinical variables were collected. Statistical analysis, including logistic regression, was performed using SPSS v28 to identify independent predictors of prolonged hospitalization. **Results:** A total of 455 pediatric patients were included, with a median age of 2 years and a median length of stay of 6 days. Prolonged hospitalization occurred in 27.5% (*n* = 125) of cases. Gender distribution did not differ significantly between groups (*p* = 0.727). Significant predictors of prolonged hospitalization included moderate-to-severe pneumonia (*p* < 0.001, OR = 9.7, 95% CI = 3.1–30.9), pneumonia complications (*p* = 0.019, OR = 15.16, 95% CI = 1.57–146.3), and underlying chronic conditions (*p* = 0.009, OR = 2.88, 95% CI = 1.3–6.4). While hypoxia, ventilatory support, and bacteremia were associated with prolonged stay, they did not emerge as independent predictors in the final multivariable model. **Conclusion:** Prolonged hospitalization in pediatric pneumonia is strongly associated with increased disease severity, complications, and chronic comorbidities. Early identification of high-risk patients may facilitate targeted management strategies, improve outcomes, and reduce healthcare burden.

## 1. Introduction

Pneumonia remains one of the leading causes of morbidity and mortality among children worldwide, accounting for approximately 15% of all deaths in children under five years of age [1,2]. Despite advances in vaccination programs and antimicrobial therapy, pneumonia continues to impose a substantial burden on healthcare systems globally, particularly in low- and middle-income countries [3,4]. Community-acquired pneumonia (CAP) is responsible for millions of pediatric hospitalizations annually, with outcomes influenced by disease severity, patient characteristics, and healthcare resource availability [5].

Standard antibiotic therapy for community-acquired pneumonia in children typically ranges from 7 to 10 days [6]. However, some children remain hospitalized beyond this period, suggesting that factors other than standard antimicrobial treatment duration contribute to prolonged stays and delayed recovery.

Hospital length of stay (LOS) serves as a critical metric for evaluating healthcare quality, resource utilization, and patient outcomes in pediatric pneumonia [7,8,9]. Prolonged hospitalization is associated with increased healthcare costs, greater risk of nosocomial infections, psychological stress for patients and families, and reduced bed availability for other critically ill children [7,8]. Understanding factors that predict extended LOS enables healthcare providers to optimize treatment protocols and implement targeted interventions to improve outcomes. Previous studies have defined prolonged hospitalization using varying thresholds, including 5, 7, 10, and 14 days [10,11,12,13]

Previous studies have identified several clinical predictors associated with prolonged hospitalization in pediatric pneumonia patients [10,14]. Disease severity, assessed through standardized scoring systems, has consistently emerged as a significant predictor of extended LOS [10,15,16,17,18]. Hypoxemia requiring supplemental oxygen, need for mechanical ventilation, bacteremia, and complications such as pleural effusion, empyema, or pneumothorax have all been linked to longer hospital stays [13,14,19]. These factors reflect the physiological burden of disease and complexity of medical interventions required for patient stabilization and recovery [6].

Beyond clinical severity markers, demographic and system-level factors also influence hospital LOS in pediatric pneumonia. Patient age, underlying chronic medical conditions such as congenital heart disease, chronic lung disease, immunodeficiency, or neurological disorders, and socioeconomic determinants have been shown to impact the duration of hospitalization [6,17,18]. Additionally, healthcare system factors including hospital capacity, staffing levels, discharge planning processes, and availability of post-discharge support services contribute to variability in LOS across institutions and geographic regions [10,14].

Despite extensive research on pediatric pneumonia outcomes in high-income countries, significant knowledge gaps persist regarding predictors of prolonged hospitalization in the Middle Eastern context, including Saudi Arabia. Regional variations in healthcare infrastructure, disease epidemiology, vaccination coverage, antimicrobial resistance patterns, and cultural practices may influence pneumonia outcomes differently than in Western populations [20]. This study, conducted at a tertiary referral center in Riyadh, aimed to identify independent predictors of prolonged hospitalization (≥10 days) in children with community-acquired pneumonia in Saudi Arabia.

## 2. Materials and Methods

### 2.1. Study Design

This retrospective cohort study analyzed clinical and demographic data from pediatric patients hospitalized with community-acquired pneumonia at King Saud University Medical City, Riyadh, Saudi Arabia, between May 2015 and March 2020.

### 2.2. Study Population

All children younger than 15 years who were hospitalized with a diagnosis of community-acquired pneumonia were eligible for inclusion. Pneumonia diagnosis was based on clinical presentation (fever, cough, tachypnea, respiratory distress) combined with radiological evidence of pulmonary infiltrates on chest radiography. Patients were excluded if they had hospital-acquired pneumonia (defined as pneumonia developing 48 h or more after hospital admission) or if they had additional primary diagnoses that could independently affect length of stay, such as acute surgical conditions or other severe systemic illnesses unrelated to pneumonia.

### 2.3. Primary Outcome

Prolonged hospitalization was defined a priori as a length of stay of 10 days or longer. This threshold was selected based on its clinical relevance, as it exceeds the typical duration of standard antibiotic therapy for community-acquired pneumonia. The objective was to identify independent predictors of prolonged hospitalization among the clinical and demographic variables collected.

### 2.4. Data Collection

Clinical and demographic data were extracted from electronic medical records using standardized data collection forms. Variables collected included patient age at admission, gender, presence of underlying chronic medical conditions (including but not limited to congenital heart disease, chronic lung disease, immunodeficiency disorders, neurological disorders, and metabolic conditions), pneumonia severity, presence of hypoxia (oxygen saturation < 93%), requirement for ventilation support (including non-invasive and invasive mechanical ventilation), development of pneumonia complications (such as pleural effusion, empyema, pneumothorax, or lung abscess), bacteremia status, and total hospital length of stay calculated from admission to discharge date.

### 2.5. Severity Assessment

The clinical severity markers used in this study were adapted from the Pediatric Infectious Diseases Society (PIDS) and Infectious Diseases Society of America (IDSA) clinical practice guidelines for the management of community-acquired pneumonia in infants and children older than 3 months [18]. Moderate-to-severe pneumonia was defined by the presence of one or more clinical features indicating respiratory compromise. These included tachypnea, dyspnea, chest retractions (suprasternal, intercostal, or subcostal), grunting, nasal flaring, apnea, altered mental status, or hypoxemia (see Table 1 for details).

### 2.6. Statistical Analysis

Descriptive statistics were calculated for all variables. Continuous variables were expressed as medians with interquartile ranges (IQR) given the non-normal distribution of hospital LOS data. Categorical variables were presented as frequencies and percentages. Patients were stratified into two groups based on the primary outcome: shorter stay (LOS < 10 days) and longer stay (LOS ≥ 10 days). Missing data were minimal and handled using complete case analysis.

Bivariate comparisons between the two LOS groups were performed using appropriate statistical tests. The Mann–Whitney U test was used for continuous variables (age), and the chi-square test or Fisher’s exact test was applied for categorical variables (gender, chronic conditions, moderate–severe disease, hypoxia, ventilation support, pneumonia complications, and bacteremia). Statistical significance was set at *p* < 0.05.

Multivariable logistic regression analysis was conducted to identify independent predictors of prolonged hospitalization. All variables from the bivariate analysis were entered into the regression model. Results were reported as odds ratios (OR) with 95% confidence intervals (CIs) and corresponding *p*-values. Model fit was assessed using the Hosmer–Lemeshow test. All statistical analyses were performed using IBM SPSS Statistics version 28.0 (IBM Corp., Armonk, NY, USA).

## 3. Results

### 3.1. Study Population and Baseline Characteristics

A total of 455 pediatric patients hospitalized with community-acquired pneumonia during the study period met inclusion criteria and were included in the analysis. The median age was 2 years in both groups (IQR: 3.9 years in the shorter stay group and 4.1 years in the longer stay group). The overall median length of hospital stay was 6 days. Among the study population, 125 patients (27.5%) experienced prolonged hospitalization defined as LOS ≥ 10 days, while 330 patients (72.5%) had shorter hospital stays of less than 10 days. Comparative clinical and demographic characteristics between patients with short and prolonged hospital stays are summarized in Table 2.

### 3.2. Demographic Characteristics

Gender distribution was balanced between groups (53.0% male vs. 51.2% male, *p* = 0.727). Patient age also did not differ significantly, with both groups having a median age of 2 years (*p* = 0.97).

### 3.3. Clinical Characteristics and Bivariate Comparisons

The presence of underlying chronic medical conditions showed a significant association with prolonged hospitalization. Among patients with shorter hospital stays, 53.9% had at least one chronic condition. In contrast, among those with prolonged hospitalization, 69.6% had a chronic condition (*p* = 0.002). Categories of chronic conditions are presented in Table 3.

Disease severity, as measured by the pneumonia severity clinical markers, was strongly associated with hospital LOS. In the shorter-stay group, 21.2% of patients had moderate-to-severe pneumonia, whereas in the prolonged hospitalization group, 72.8% had moderate-to-severe disease (*p* < 0.001).

Hypoxia was significantly more frequent among patients with prolonged hospitalization (91.2% vs. 62.7%, *p* < 0.001). The need for ventilation support, including both non-invasive and invasive mechanical ventilation, differed markedly between groups. In the shorter-stay group, 10.4% of patients required ventilatory support, whereas in the prolonged hospitalization group, 34.4% required ventilation support (*p* < 0.001)

Pneumonia complications (mainly pleural effusion with or without necrotizing pneumonia) were significantly associated with prolonged hospitalization. In the shorter-stay group, 0.3% of patients developed complications, whereas in the prolonged hospitalization group, 12.0% developed complications (*p* < 0.001)

Bacteremia status also showed a significant association with hospital duration. In the shorter-stay group, 3% of patients had positive blood cultures, whereas in the prolonged hospitalization group, 12.0% were bacteremia-positive (*p* < 0.001).

### 3.4. Multivariate Logistic Regression Analysis

**Figure 1:**
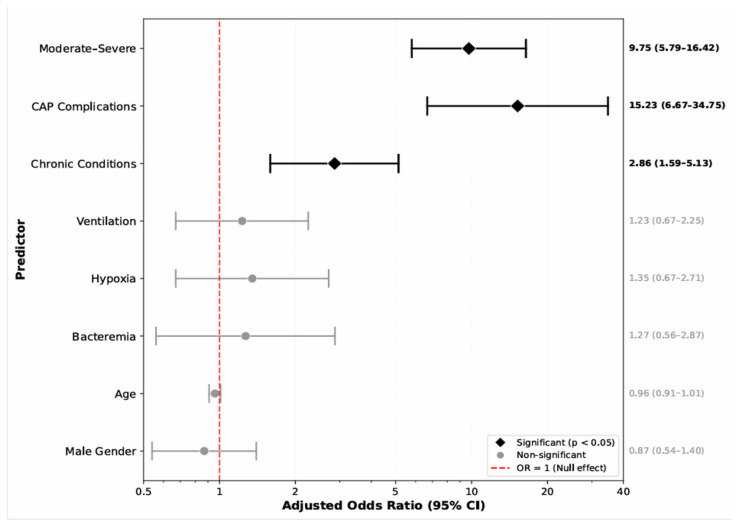
Predictors of prolonged hospitalization (LOS ≥ 10 Days) in children with community-acquired pneumonia.

Multivariable logistic regression analysis identified three independent predictors of prolonged hospitalization (Table 4, Figure 1). Pneumonia complications were the strongest predictor, with patients having 15.17 times higher odds of prolonged hospitalization (OR = 15.17, 95% CI: 1.57–146.35, *p* = 0.019). Moderate-to-severe disease was associated with nearly 10-fold increased odds of extended hospital stay compared to mild disease (OR = 9.75, 95% CI: 3.08–30.91, *p* < 0.001). Underlying chronic medical conditions conferred almost three times the odds of prolonged hospitalization (OR = 2.88, 95% CI: 1.30–6.39, *p* = 0.009). Other variables—gender, age, hypoxia, ventilation support, and bacteremia—did not remain significant after adjustment.

## 4. Discussion

This study examined predictors of prolonged hospitalization in children with community-acquired pneumonia at a tertiary care center in Saudi Arabia. Among the cohort, 27.5% of children experienced a prolonged length of stay (≥10 days), with independent predictors including moderate-to-severe disease, underlying chronic medical conditions, and pneumonia-related complications. This proportion aligns with previously reported rates of 25–43% [12,14,16,21].

Underlying chronic medical conditions were an independent predictor of prolonged hospitalization in this cohort, consistent with existing evidence demonstrating that children with comorbidities are at higher risk for severe respiratory infections and delayed recovery [17,22]. The nearly threefold increase in odds observed here aligns with findings from Kebede et al., who reported an odds ratio of 2.64 for prolonged hospitalization among children with chronic conditions [18].

Pneumonia-related complications, including pleural effusion, empyema, and lung abscess, were the strongest independent predictors of prolonged hospitalization, demonstrating an approximately 15-fold increase in odds. While these complications were infrequent, leading to wide confidence intervals, the finding is consistent with existing research [13,19]. McClain et al. reported that moderate-to-large pleural effusions were associated with prolonged hospitalization (OR 2.6) [19]. These complications significantly increased diagnostic and treatment demands, often requiring invasive procedures and prolonged antibiotic therapy [6,13].

Moderate–severe disease was a strong independent predictor of prolonged hospitalization. This finding is consistent with previous studies demonstrating that severe pneumonia is associated with longer hospital stays in children [17,21,22]. Gonapaladeniya et al., using the WHO classification, reported an odds ratio of 22.1 for prolonged hospitalization among children with severe disease [21]. A similar association was observed in this study, despite the use of a different PIDS/IDSA moderate–severe disease definition, suggesting the robustness of disease severity as a predictor across settings.

Clinical components of a moderate–severe disease score—such as hypoxia, tachypnea, and chest retractions—have also been linked to prolonged hospitalization [11,16,21]. Kuti et al. found hypoxia to be predictive of prolonged LOS (OR 2.22) [14]. In the present study, although hypoxia and need for ventilatory support were more frequent in the prolonged hospitalization group, they did not remain statistically significant independent predictors after multivariable adjustment. Furthermore, collinearity was observed among several severity-related variables, including hypoxia, need for ventilatory support, and the overall severity classification. Despite this statistical overlap, these variables were retained in the multivariable model due to their clinical relevance and established importance in pneumonia outcomes. Notably, only moderate-to-severe disease emerged as a strong independent predictor of prolonged hospitalization after adjustment, suggesting that the composite severity classification captures the predictive strength of its individual clinical components.

Jakhar et al. identified bacteremia as a strong predictor of prolonged hospitalization (OR 15.2) [16]. In contrast, bacteremia was not a significant independent predictor in the present study. This discrepancy may be explained by differences in the definition of prolonged LOS, as Jakhar et al. used a ≥5-day threshold compared to the ≥10-day cutoff applied here.

No significant associations were found between age or gender and prolonged LOS in either bivariate or multivariable analyses. This suggests that these demographic factors do not substantially influence LOS in the studied age range. However, very young infants and adolescents may represent distinct subpopulations warranting separate investigation.

Several limitations warrant consideration. The single-center retrospective design may limit generalizability. Microbiological data, including viral etiologies and antimicrobial resistance patterns, were not systematically captured. The dichotomization of LOS at 10 days, while clinically meaningful, may reduce statistical power compared to analyzing LOS as a continuous variable. Additionally, unmeasured factors such as socioeconomic determinants and discharge processes may have influenced hospital duration. As a retrospective electronic medical record-based study, potential coding or documentation bias may affect data completeness and accuracy.

These findings have several implications for the clinical management of pediatric pneumonia. First, identifying risk factors for prolonged LOS can support early risk stratification at admission, enabling timely subspecialty referral, enhanced monitoring, and informed discussions with families. Second, awareness of these predictors may help hospitals plan for resource allocation, including bed management and discharge processes. Third, targeted interventions for modifiable risk factors such as prompt escalation of antibiotics, early detection of complications, and optimization of chronic disease management may help reduce LOS without compromising care.

Future studies should include larger, multi-center cohorts across diverse healthcare settings to improve generalizability. Additional variables warrant investigation, including microbial etiology, antimicrobial resistance patterns, socioeconomic factors, and discharge planning processes. Comparative studies evaluating different pneumonia severity markers and their ability to predict prolonged hospitalization would help identify optimal risk stratification tools. Disease-specific analyses examining outcomes in children with individual chronic conditions (e.g., congenital heart disease, neuromuscular disorders, chronic lung disease) compared with healthy children may further refine prediction models and guide tailored management strategies. Development of early risk prediction tools and quality improvement initiatives could help streamline care and reduce unnecessary prolonged hospitalizations.

## 5. Conclusions

Pneumonia severity, the presence of complications, and underlying chronic medical conditions independently predicted prolonged hospitalization in children with community-acquired pneumonia. With more than one-quarter of patients hospitalized for 10 days or longer, these findings highlight the substantial burden of extended hospital stays and underscore the need for targeted strategies to reduce length of stay.

## Figures and Tables

**Table 1:** Criteria for respiratory distress in children with pneumonia (PIDS/IDSA).

Signs of Respiratory Distress
Tachypnea (respiratory rate, breaths/min): • Age 0–2 months: >60 • Age 2–12 months: >50 • Age 1–5 years: >40 • Age > 5 years: >20 Dyspnea Retractions (suprasternal, intercostal, or subcostal) Grunting Nasal flaring Apnea Altered mental status Pulse oximetry measurement <90% on room air

**Table 2:** Clinical characteristics of prolonged hospitalization and shorter stay groups.

Variable	Shorter Stay *n* = 330	Prolonged Hospitalization *n* = 125	*p* Value
Male sex, *n* (%)	175 (53.0)	64 (51.2)	0.73
Age, median (IQR), years	2 (3.9)	2 (4.1)	0.97
Chronic conditions, *n* (%)	178 (53.9)	87 (69.6)	<0.01
Moderate-to-severe disease, *n* (%)	70 (21.2)	91 (72.8)	<0.01
Oxygen supply, *n* (%)	207 (62.7)	114 (91.2)	<0.01
Ventilation support, *n* (%)	34 (10.3)	43 (34.4)	<0.01
Pneumonia complications, *n* (%)	1 (<1)	15 (12.0)	<0.01
- Empyema, *n*	1	9	
- Necrotizing pneumonia, *n*	0	2	
- Empyema with necrotizing pneumonia, *n*	0	4	
Bacteremia, *n* (%)	10 (3.0)	15 (12.0)	<0.01

**Table 3:** Chronic conditions in categories.

Clinical Category	Included Conditions	*n* (%)
Genetic and Congenital Disorders	Genetic disorders; congenital anomalies; congenital respiratory anomalies; inborn errors of metabolism	72 (15.8)
Cardiac Disorders	Congenital heart disease	8 (1.8)
Neurological Conditions	Neurodevelopmental disorders; neuromuscular disorders; neurological diseases	62 (13.6)
Respiratory Disorders	Pulmonology/respiratory diseases	49 (10.8)
Hematologic Disorders	Hemoglobinopathies; hematologic disorders	13 (2.9)
Immunologic and Inflammatory Disorders	Immune dysregulation/immune defects; allergic/atopic disorders; rheumatologic/vasculitic disorders	13 (2.9)
Other Systemic Disorders	Endocrine; hepatobiliary; oncologic disorders	6 (1.3)

**Table 4:** Summary of logistic regression.

Variable Category	Coefficient	S.E.	*p* Value	Odds Ratio	95% C.I. for Odds Ratio	
					Lower	Upper
Gender	0.072	0.323	0.824	1.075	0.571	2.024
Age	−0.006	0.049	0.896	0.994	0.902	1.094
Moderate–Severe Disease	2.277	0.589	<0.001	9.752	3.077	30.908
Hypoxia	0.426	0.456	0.350	1.531	0.626	3.745
Ventilation support	−0.107	0.618	0.862	0.898	0.268	3.013
Bacteremia	0.572	0.730	0.433	1.772	0.424	7.406
Pneumonia complication	2.719	1.157	0.019	15.166	1.572	146.349
Underlying condition	1.057	0.407	0.009	2.878	1.296	6.387

Note: The Hosmer–Lemeshow test showed good fit (*p* = 0.2).

## Data Availability

The data that support the findings of this study are available from the corresponding author upon reasonable request. The data are not publicly available due to due to patient privacy considerations and institutional data governance policies.

## Abbreviations

The following abbreviations are used in this manuscript:

CAP	Community-acquired pneumonia
LOS	Length of stay
KSUMC	King Saud University Medical City
PIDS	Pediatric Infectious Diseases Society
IDSA	Infectious Diseases Society of America
IQR	Interquartile ranges
OR	Odds ratios
CI	Confidence intervals
IRB	Institutional Review Board
